# Intent to migrate among nursing students in Uganda: Measures of the brain drain in the next generation of health professionals

**DOI:** 10.1186/1478-4491-6-5

**Published:** 2008-02-12

**Authors:** Lisa Nguyen, Steven Ropers, Esther Nderitu, Anneke Zuyderduin, Sam Luboga, Amy Hagopian

**Affiliations:** 1 School of Medicine, University of Washington, Seattle, USA; 2 Department of Nursing, Aga Khan University School of Nursing, Kampala, Uganda; 3 Department of Medicine, Makerere University, Kampala, Uganda; 4 Department of Health Services, University of Washington School of Public Health, Seattle, USA

## Abstract

**Background:**

There is significant concern about the worldwide migration of nursing professionals from low-income countries to rich ones, as nurses are lured to fill the large number of vacancies in upper-income countries. This study explores the views of nursing students in Uganda to assess their views on practice options and their intentions to migrate.

**Methods:**

Anonymous questionnaires were distributed to nursing students at the Makerere Nursing School and Aga Khan University Nursing School in Kampala, Uganda, during July 2006, using convenience sampling methods, with 139 participants. Two focus groups were also conducted at one university.

**Results:**

Most (70%) of the participants would like to work outside Uganda, and said it was likely that within five years they would be working in the U.S. (59%) or the U.K. (49%). About a fourth (27%) said they could be working in another African country. Only eight percent of all students reported an unlikelihood to migrate within five years of training completion. Survey respondents were more dissatisfied with financial remuneration than with any other factor pushing them towards emigration. Those wanting to work in the settings of urban, private, or U.K./U.S. practices were less likely to express a sense of professional obligation and/or loyalty to country. Those who have lived in rural areas were less likely to report wanting to emigrate. Students with a desire to work in urban areas or private practice were more likely to report an intent to emigrate for financial reasons or in pursuit of country stability, while students wanting to work in rural areas or public practice were less likely to want to emigrate overall.

**Conclusion:**

Improving remuneration for nurses is the top priority policy change sought by nursing students in our study. Nursing schools may want to recruit students desiring work in rural areas or public practice to lead to a more stable workforce in Uganda.

## Background

Like sub-Saharan African countries, Uganda's 29 million people face huge health challenges, including HIV, malaria, TB, maternal mortality (880 per 100,000 live births) and child mortality (79 per 1000 live births) [1]. Uganda spends about 1.3 percent of its gross national product on health, or about $23 per person per year. We conducted a study of nursing student career intentions in Uganda to gain an understanding of factors that could encourage nurses to practice in settings where they would most contribute to addressing this country's large health challenges.

Regardless of the ratio of nurses to the population, most countries around the world have declared themselves to be in need of additional nurses. Among those claiming a shortage is the United States (U.S.), which has 773 nurses to 100,000 population, and Uganda, with a dismal six nurses to 100,000 population [2]. Nurses in low-income countries are migrating to wealthier countries in search of better salaries, improved working conditions, and more opportunities for further training, resulting in a "brain drain." The predicted additional nurse requirements of the developed world are large enough to deplete the supply of qualified nurses throughout the developing world [3].

There is an established connection between adequate health worker staffing levels and positive care outcomes. The Joint Learning Initiative examined various measures of health care outcomes in countries around the globe, compared those outcomes to the number of health workers in the population, and determined that countries need at least 2.5 health workers per thousand population to achieve minimal health care coverage [4]. Sub-Saharan Africa needs 600,000 additional nurses to meet the average density for low-income countries, a goal unlikely to be achieved with the continued exodus of nurses from African countries [2]. Africa, with 24 percent of the global burden of disease, employs only three percent of all health professionals [5]. Nurses in Africa are arguably the most important health care workers available in most sub-Saharan nations, as they perform a broad range of tasks and are often working in settings where no other health workers, including physicians, are available [6]. Further exacerbating health disparities, African health professionals, not unlike their counterparts worldwide, prefer to work in urban areas over rural areas [7].

While there have been numerous studies on the exodus of nurses from Africa, these studies have primarily focused on the views of and the push/pull factors affecting nurses already in practice. Push factors for health workers include poor remuneration and conditions of service, civil unrest, lack of opportunities for postgraduate training, feelings of lack of respect/value placed in health workers by country/system, and concern about poor governance and management of the health system [3,8-14]. Pull factors include opportunities for further training and career advancement, greater financial rewards and improved working conditions, availability of posts, job security and job satisfaction [3,9,11-14]. In addition, factors such as loyalty to country, sense of professional and pride, and expectations of depression have been mentioned in literature, but not yet studied [8]. There has not yet been a published study that elucidates nursing students' intentions and perceptions of emigration while they are still pursuing their educational training. Exploring students' conceptions allows a fresh plane of analysis and a new avenue of possible interventions to this problem. Information on the views of the next generation of nursing professionals can help shape policy at training institutions as well as at the governmental level.

This paper was intended to explore students' views on the factors that will influence their future practice locations, along three dimensions: rural/urban; public/private; and in Uganda, another African country or abroad. We created a conceptual framework illustrating the profiles of qualities associated with various practice preferences, based on our findings. This contribution to the literature is made in the interest of informing school admissions policies, scholarship policies, and approaches to training.

## Methods

Two of Uganda's 32 nursing schools participated in this study, Makerere Medical School and Aga Khan University. Both are located in the capital city, Kampala. In the 2005/2006 academic year, 168 nursing students were enrolled at Aga Khan, a private university, while 348 were enrolled at Makerere University, the largest public university in the country. These universities typically admit nurses seeking to upgrade their training from a lower-level "enrolled" status to a "registered nurse" and/or Bachelor of Science in Nursing status. Students in both institutions typically pay only a fraction of the cost of their education, as the institutions are both highly subsidized and students often receive scholarships. The study was conducted by two University of Washington medical students and their faculty preceptor during July 2006 using convenience sampling methods. Participation was voluntary and anonymous, including nursing students at any level of their education. The written survey consisted of 68 questions, most of them with closed-ended (five-point Likert) answer scales. Two focus groups were also conducted at Aga Khan University, consisting of eight to twelve volunteers each.

Questionnaires measured the influence of various factors associated with the intent to migrate by health care workers, identified from the literature. These included demographics, stability and safety of the country, finances, sense of professional pride and obligation, future plans, and outlook of working conditions. Outcome variables for emigration included questions pertaining to emigration to the U.K., U.S., or another African country. Outcome variables for desired type of practice were questions relating to preference for working in urban or rural areas and public or private practice. All questionnaires were double-entered using Epi Info and analyzed statistically using SPSS v 14.0. Student's T-tests were performed, with significance defined as p value ≤ 0.05. On the five-point scale, "4" or "5" were grouped together to create an "agree" category for many of the questions. An answer of "3" was considered to be neutral.

Focus groups were not tape recorded, but extensive notes were taken by hand and entered into a computer within 24 hours.

Human subjects approval was obtained from Ugandan National Council of Science & Technology, both participating nursing schools, and received an exemption from the University of Washington Human Subjects due to minimal risk categorization.

## Results

We collected 158 questionnaires, the majority (60%) from Aga Khan. Table 1 summarizes the characteristics of the nursing students participating in this study. The majority were female (82%), with an average age of 32. Of these, 57 percent were in the process of attaining their diploma while 25 percent were earning their baccalaureate. About a third (35%) were in first year, 16 percent in second year, and 36 percent were in the third year of their programs. Non-respondents to each question were disregarded.

**Table 1:** Respondent personal characteristics

		**Number of respondents**	**Percent of respondents**
**School**	Aga Khan	95	60.1%
	Makerere	44	27.8
**Program**	Diploma	90	57.0
	Bachelor	39	24.7
**Year in school**	Year 1	26	34.8
	Year 2	57	16.5
	Year 3	20	36.1
**Gender**	Female	130	82.3
	Male	16	10.1
**Marital status**	Single	70	44.3
	Married	74	46.8
**Area lived before age 17**	City	61	38.6
	Rural	86	54.4
**Ever live in rural area?**	Yes	130	82.3
	No	18	11.4
**Non-government primary school**	Yes	73	46.2
	No	77	48.7
**Father-tertiary education**	Yes	102	64.6
	No	49	31.0
**Mother-tertiary education**	Yes	66	41.8
	No	85	53.8
**From which region?**	Northern	22	13.9
	Southern	4	2.5
	Western	30	19.0
	Eastern	27	17.1
	Central	64	40.5
**Age Avg = 31.6**	Age 20–30	51	32.3
	Age 31–40	54	34.2
	Age 41–50	15	9.5
	51 and over	3	1.9

**Average number of children: **1.8 kids

It is a limitation of the study that we do not have data on the level of nurse training in which each student was enrolled, nor the percentage of students who had government-subsidized education. We also were unable to conduct focus groups at Makerere for logistical and scheduling reasons, so our focus group data come only from the private university.

### Quantifying push/pull factors: finances, safety and stability, sense of professionalism

Table 2 summarizes participants' general views of Uganda's safety and stability, the financial prospects for nursing professionals, and sense of professionalism. Only 30 percent of nursing student respondents thought Uganda had been stable over the last five years and 61 percent of respondents would prefer to move to a more stable country. While 39 percent of participants thought it was safe to work in urban Uganda, only 19 percent thought it was safe to work in rural Uganda. Financial satisfaction as a nurse was perceived to be highest in the U.S. or Canada (94% of respondents agreed), followed by Europe (89%), another African country (41%), then lastly Uganda (5%). When discussing the role of the nursing profession in their country, 88 percent of participants thought they made a difference in the country's well-being and that nurses were role models for other people. When asked about the obligations of students to "repay" their free, government-sponsored education, about 70 percent of the students said if the country had paid for a nurse's education, the nurse should stay in the country. Approximately half of the participants reported a desire to move abroad since childhood or before beginning nursing school.

**Table 2:** Frequency/means summary table of general opinions

	N	Mean	% positive opinion*
**Stability & safety**			

I would move to another country if the country is more stable.	143	3.59	60.8
How safe is it for a nurse to work in urban Uganda?	150	3.29	39.3
How stable will the country be over the next five years?	149	2.99	35.6
How stable has the country been over the last five years?	153	2.90	30.1
How safe is it to work as a nurse in rural Uganda?	145	2.28	19.3

**Finances**			

How financially satisfying is working as a nurse in the U.S./Canada?	149	4.62	94.0
How financially satisfying is working as a nurse in Europe?	147	4.36	89.1
I would move to another country if the financial offer is better.	154	4.34	83.8
How financially satisfying is working as a nurse in other African countries?	145	3.19	41.4
How financially satisfying is working as a nurse in Uganda?	153	1.65	5.2

**Stability vs. finances**			

I would move to another country if the financial offer is better but the country is less stable.	144	2.47	27.1
I would move to another country if the financial offer is worse but the country is more stable.	149	2.34	23.5

**Sense of profession**			

As a nurse, I am a role model for other people.	153	4.52	88.2
As a nurse, I make a big difference in a country's well-being	156	4.54	87.8

**Responsibility to country**			

If a country has paid for a nurse's education, the nurse should stay in the country to help the people.	155	3.94	69.7
If a nurse has paid for his/her own education, he/she is not required to stay in that country.	153	3.05	42.5

**Desire to move**			

Moving abroad has been a desire of mine since I was a kid.	152	3.64	55.9
Moving abroad has been a desire of mine before I started nursing school.	155	3.34	51.0

* "positive opinion" means answered 4 or 5 on a scale of 1 through 5.

### Job outlook

Table 3 compares students' opinions on expected working conditions in Uganda and abroad. There were no statistically significant differences between how students viewed their working situations if they were to work in Uganda as compared to abroad, but the data in most cases suggested a more satisfactory set of arrangements abroad, especially in the areas of overall job satisfaction and ability to support one's family. Students reported similar expectations in having family/social support, finding a job matched to skill, and being able to increase in job rank. Almost half (46%) said they would likely experience racism while working abroad. About a third (34%) thought it likely they would experience depression if working abroad while 30 percent thought it likely if working in Uganda.

**Table 3:** Students' outlooks on working conditions in Uganda and abroad

	**If you were to work in Uganda, how likely would you:**		**If you were to work abroad, how likely would you:**	
	
	Mean	% likely*	Mean	% likely*
Have control of your practice?	3.91	70.8%	4.00	72.1%
Find a job matched to your skill?	4.01	70.4	4.02	77.9
Be able to increase your job rank?	3.86	66.4	4.12	76.9
Have family/social support?	3.59	62.2	3.58	60.0
Experience occupational risk?	3.52	54.6	3.45	56.4
Be able to provide for your family?	3.35	46.9	4.35	83.2
Be satisfied with your job?	3.31	44.1	4.18	83.0
Experience depression?	2.60	29.8	2.77	33.8
Experience racism?	N/A	N/A	3.14	46.4

*Answered 4 or 5 on a scale of 1 through 5 (1 = unlikely, 5 = very likely)

In the focus groups, nursing students revealed the following dissatisfactions with working conditions in Uganda:

#### • Too little pay

Average nursing pay of 200,000 Ugandan shillings per month (the equivalent of $115) was not enough to meet basic needs. A minimum of 500,000 Ugandan shillings ($290) was suggested as a starting point to begin to meet basic needs, although some in the group argued that amount was insufficient to compensate for the risks and work involved in typical patient ratios. One participant said, "When you look at the nurses who are teaching you, they have nothing to show. They are not role models actually. If someone graduated in 1981, and doesn't have a house of her own or a good bank statement – why should I want to be like them? I want to go out and make money, and send money home to put my kids in the best schools in Uganda."

#### • Inadequate equipment and supplies

Often there were no gloves to protect nursing staff from body fluids and if working in a TB unit, there were no protective masks.

#### • Poor benefits

Nurses are often not insured or provided with health insurance by their employers.

#### • Not enough public-sector jobs for nurses

Nursing students reported many trained nurses are now working in supermarkets or as bar maids. The private not-for-profit sector (generally operated by Catholic, Protestant or Muslim Medical Bureaus) provides jobs for nurses, but some nurses said they are exploited there: "Patients in private hospitals are frustrating. They don't respect you or value you as a nurse."

#### • Nurses treated badly

Many nursing students felt doctors often make nurses take blame for the doctors' own mistakes. They felt they had little job protection and they also cannot afford to hire lawyers to protect their rights. A nurse currently in the workforce reported that her employer does not forward her contributions to the National Social Security (retirement) Fund. Nurses are afraid to complain, because they could easily lose their jobs: "When you complain, they tell you to get out."

### Future work intentions

Table 4 summarizes the students' plans for employment. Most (70%) of the participants would like to work outside Uganda, and said it was likely that within five years they would be working in the U.S. (59%) or the U.K. (49%). About a fourth (27%) said they could be in another African country. Only eight percent of all students reported an unlikelihood to migrate within five years of training completion. Approximately three in four (76%) reported they would return to Uganda if they were to work abroad.

**Table 4:** Frequency/means summary table of future plans

	N	Mean	% likely*
**Where would you like to work after you complete all of your training?**			

Urban	121	4.21	80.2
Rural	114	2.54	28.9
Private	110	3.37	58.2
Public	115	3.72	83.5
In Uganda	121	3.82	63.6
In another country	126	3.94	69.8

**What are the chances you will leave Uganda to work as a nurse within five years of completing your training?**			

In the U.K.?	138	3.26	49.3
In another African country?	119	2.54	26.9
In the U.S.?	131	3.53	58.8
Somewhere else?	121	2.69	34.7

**Do you plan to seek post-graduate training?**			

Yes	138	N/A	N/A
No	8	N/A	N/A

**If given the opportunity to go abroad for further training, would you go?**	156	4.74	91.7

**If you were to work abroad, how likely would you return back to Uganda?**	120	4.16	76.1

*Answered 4 or 5 on a scale of 1 through 5 (1 = unlikely, 5 = very likely)

A large majority (80%) said they would like to work in urban areas after completing their training, while only 29 percent would prefer work in rural areas. Working for the public sector was more favourable than the private sector (84% vs. 58%).

In focus groups, students favoured migration to the U.S. over the U.K. because they perceived gaining entrance to the U.K. to be too competitive. Most students learned about emigration opportunities from friends and colleagues who had already emigrated. They stated that emigration information was not readily available or accessible. One student even reported she had misrepresented herself at the U.K. embassy to get information about a work permit visa because she felt officials would disapprove of her emigration if they knew she was a nurse.

Nursing students expressed wariness over companies that promised opportunities abroad, citing a recent incident in which a government official was using the government office to recruit nurses for job opportunities abroad, collecting money from the nurses, but never providing the job opportunities. The students claim that the incident has been reported, but the government has done nothing about it yet.

Students reported the importance of family as a reason to stay in Uganda: "If pay is good, then I don't think nurses will think of leaving. People want to stay with their families, but then they sacrifice to go."

### Urban-inclined nursing student versus rural-inclined nursing student

Those inclined to work in rural areas would not be motivated to emigrate out of concern about country stability or financial incentives, in direct contrast to those intending to work in urban areas (p ≤ 0.05) (see Table 5). Rural-bound nursing students were the only ones to say pay in the U.S. or Canada would bring low satisfaction (p ≤ 0.01), and to register a higher sense of professional pride, believing that they were important role models (p ≤ 0.05). Ironically, they were also the sub-group that expected to experience depression when working in Uganda (p ≤ 0.02). Demographically, rural-bound nursing students tended to be older (p ≤ 0.04).

**Table 5:** Profile comparison between nursing students wanting to work in an urban vs. rural area

**Profile: wants to work in an urban area**	**Profile: wants to work in a rural area**
***80.2% ****responded "highly likely" to work in an URBAN area*	***28.9% ****responded "highly likely" to work in a RURAL area*
• Would emigrate for financial reasons and country stability • Believes pay in U.S./Canada brings high satisfaction • Has desired to move abroad since before starting nursing school • Would work in private practice	• Would not emigrate for financial reasons or country stability • Believes pay in U.S./Canada brings low satisfaction • Believes he/she is a role model for other people • Would not work in private practice or • Would not work in another country • Expects depression when working in Uganda • More likely to be an older student

Urban-bound students were associated with an interest in private practice and had wanted to emigrate since before starting nursing school (p ≤ 0.01). They would emigrate for financial incentives and country stability (p ≤ 0.01).

### Private-practice nursing student versus public-practice nursing student

Like their rural-bound counterparts, nursing students seeking work in public practice also believed they were role models for other people (p ≤ 0.01) (see Table 6). In addition, public-minded students also believed nursing students should stay in country if the country paid for the nurse's education (p ≤ 0.02). Although these students reported wanting to work in Uganda, they were also associated with wanting to move abroad since childhood (p ≤ 0.01). Country stability was more important than financial incentives for those wanting to work in public practice (p ≤ 0.05). Demographically, those wanting to work in public practice had more children (p ≤ 0.04).

**Table 6:** Profile comparison between nursing students wanting to work in private vs. public practice

**Profile: wants to work in a private practice**	**Profile: wants to work in a public practice**
***58.2% ****responded "highly likely" to work in private sector*	***83.5% ****responded "highly likely" to work in public sector*
• Would emigrate to a more stable country • Would emigrate for financial reasons • Believes pay in U.S./Canada brings high satisfaction • Expects job dissatisfaction when working in Uganda	• Would emigrate to a more stable country even if financial offer is worse • Believes he/she is a role model for other people • Believes nursing students should not emigrate if country has paid for the nurse's education • Would like to work in Uganda • Has desired to move abroad since childhood • Expects to increase in job rank if working abroad • Plans to continue with post-graduate training • Attended a governmental primary school • Has 1 or more children

For those wanting to work in private practice, country stability and finances were both important factors that would encourage emigration (p ≤ 0.01). Those wanting to work in private practice also were more likely to expect dissatisfaction with their job in Uganda (p ≤ 0.02).

### Students preferring to work in the U.K., U.S., or African country

Nursing students attracted to the U.K. had the least affinity for working in rural areas, having not lived in a rural area prior to age 17, and expecting it to be unsafe to work in rural Uganda (p ≤ 0.05) (see Table 7). They also were more likely to anticipate a favourable working situation abroad, including control over their practice, being able to provide for their family, and having family/social support (p ≤ 0.01). U.K.-bound students also reported a desire to move abroad since childhood (p ≤ 0.01).

**Table 7:** Profile comparison of nursing students wanting to emigrate to the U.K., U.S., or another African country within five years of graduation.

**U.K.-bound characteristics**	**U.S.-bound characteristics**
***49.3% ****responded "highly likely" to emigrate to U.K*. • Believes it is unsafe to work in rural Uganda • Would emigrate to a more stable country • Believes pay in Europe brings high satisfaction • Has desired to move abroad since childhood • Would not want to work in a rural area • Expects control over practice, having family/social support, providing for family if working abroad • Is not from the central (urban) region of Uganda Did not live in a rural area prior to age 17	***48.8% ****responded "highly likely" to emigrate to U.S*. • Would emigrate to a more stable country even if financial offer is

**Overlapping characteristics between U.S.- & U.K.-bound**	**African country-bound characteristics**

• Would emigrate for financial reasons • Expects job matched to skill, able to increase in rank, & job satisfaction if working abroad • Expects occupational risk if working abroad • Had mother who completed tertiary education	***26.9% ****responded "highly likely" to emigrate to another African country* • Believes pay in other African countries brings high satisfaction • Believes he/she is a role model for other people • Expects not to have control over practice of working abroad • Male gender

Students seeking to move to the U.S. expressed a high value for stability, even if the financial incentives were worse (p ≤ 0.04).

Expectations shared by both groups of students intending to emigrate to the U.K. or U.S. were of having a positive outlook of working conditions abroad, including finding a job matched to skill, ability to increase in rank, and job satisfaction (p ≤ 0.01). These students also expected occupational risk if working abroad (p ≤ 0.05). Students seeking to move to the U.K. or U.K. had mothers who had completed tertiary education (p ≤ 0.05).

Nursing students intending to work in another African country believe they are role models for other people (p ≤ 0.02) and are more likely to be male. They are attracted to higher pay in other African countries (p ≤ 0.01) and believe that they would not have control of their practice if working abroad (p ≤ 0.05). No particular African country destination dominated the answers to this open-ended question.

## Discussion

### Push/pull factors

This study identified financial remuneration as more important to student nurses than all the other push/pull factors we measured. This concurs with literature suggesting that compensation constitutes the most basic influence on retention of health professionals [7,15]. While 30 percent of respondents had a positive opinion about Uganda's safety and stability, only five percent of respondents thought that working as a nurse in Uganda was financially satisfying. Ugandan nurses earn less than $100 per month, compared to an average $3000 in the U.S [16]. In a 2004 report, Uganda's nursing wages were reported to be the lowest among a set of comparable sub-Saharan countries [16].

Generally, the threat from civil unrest, public protests, demonstrations and political violence is gauged to be low in Uganda, with the exception of northern Uganda, where the Lord's Resistance Army operates [17]. The average age of students, more than 30 years, would date them to having been reared during the 1970s, when Idi Amin's rule of Uganda led to chaos and prosecution of intellectuals; this may influence their sense of the nation's stability and safety.

Outlook on working conditions in Uganda compared to those abroad were not statistically significant, although in most cases suggested a more satisfactory set of arrangements abroad. Students had similar expectations about being able to control their practice, find a job matched to their skill level, experience depression as well as having family and social support in both locations. This suggests that decreasing the pay gap between Uganda and other countries would be more immediate in stemming nursing dissatisfaction than improving working conditions because students' expectations of working conditions in Uganda and abroad were comparable.

### Intent to migrate

We found 70 percent of nursing students expressed an intent to migrate out of Uganda. The percentage of nursing students desiring to emigrate is substantially higher than the rate reported for established health workers by the World Health Organization's Africa Regional Office (27%) [18]. Students are, of course, a more mobile and younger population, and would be expected to report different intentions than established health workers. Although the intent to migrate was high, three in four (76%) reported they would return to Uganda after working abroad. This again seems to imply that finances are the main motivation for emigration. If differences in working conditions and country stability were the main motivators, we would expect that students would not want to return since these factors would likely remain unchanged in the time they were away from Uganda.

Students in our study, as revealed by both the questionnaire and the focus groups, reported having a stronger desire to emigrate to the U.S. or Canada than to the U.K., which would be a new direction for most of Uganda's health worker migrants. Traditionally, the trajectory for most migrants has been to the U.K. rather than the U.S. In our study, students reported they perceived entrance into the U.S. to be easier than the U.K. because there were already too many foreign nurses in the U.K.

These findings, coupled with provisions under consideration by the U.S. Congress, which would lift restrictions on nurse migrants to the U.S., could spell a significant new exodus for Ugandan nurses, especially new graduates [6]. This is of particular concern because the U.S., unlike the U.K., has yet to develop ethical recruitment guidelines that limit the aggressive recruitment of health workers from low-income countries. Since 1998, foreign-trained nurse entrants to the U.S. nurse sector have increased at a rate faster than that of U.S.-educated new nurses [3]. This has not been the result of a lack of interest on the part of would-be nursing students in the U.S., as more than 11,000 qualified students were denied admission to nursing schools in 2003 as the result of limited capacity [3].

### Future practice locations

It was not a surprise to discover that students preferred to work in urban over rural areas (80% versus 29%). Uganda Ministry of Health statistics report that 64 percent of all nurses and midwifery professional cadres work in Uganda's central (most urban) region, where only 27 percent of the population resides [19]. However, student preference for the public sector over the private sector (84% vs. 58%) was surprising as it is different from developed countries where health workers seem to prefer to work in the private sector [20]. Our nursing students expressed a clear preference for public sector jobs, and expressed dismay that some of their fellow nurses were working in unrelated jobs, such as bar maid. A country cannot hope to retain nurses if there are not enough jobs to employ them. There are no data on the actual percentage of nurses now working in non-nursing jobs.

### Factors influencing preference for future practice locations

**Figure 1:**
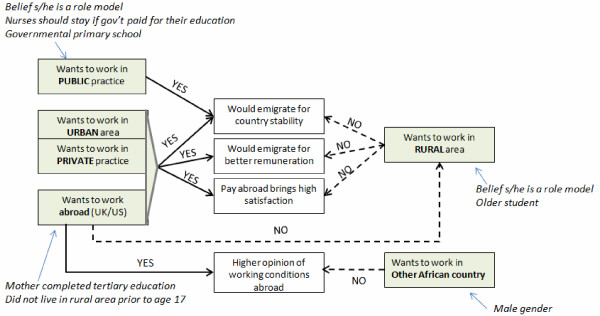
**Conceptual model of factors influencing Ugandan nursing students' practice intentions**. Source of data for model: Surveys and focus groups of nursing students at Uganda's Makerere and Aga Khan Universities; July, 2006.

When analyzing factors influencing students' preferences for future practice locations, a pattern emerged, separating those who intended to emigrate and those who did not. From the findings in this study, we created a conceptual framework to illustrate the career intentions of student nurses, based on correlations with student attitudes and expectations. See Figure 1. The most basic division is that students wanting to work in urban areas, private practice or abroad would emigrate for any number of reasons, including financial reasons and country stability, while those wanting to work in rural areas do not express a desire to emigrate. Those wanting to work in public practice would emigrate only for country stability. Students wanting to emigrate abroad would not wish to work in rural areas.

On a more complex level are correlations of practice location with personality factors, demographics and personal opinions. A sense of professional pride and loyalty to country were factors that also separated those who wished to work in rural areas from those wanting to work in urban areas, private practice or abroad. Students wanting to work in public practice or another African country were similarly associated with a sense of professional pride and loyalty to country. Those wanting to work abroad were the only sub-group of students associated with having a more positive outlook of working conditions abroad and had mothers who had completed tertiary education, suggesting a higher socioeconomic status. Rural-bound students tended to be older, which might suggest that they have greater family responsibilities and therefore a lower desire for emigration in contrast to someone who is younger [7]. Of all sub-groups, rural-bound students also expected to experience depression if they stayed to work in Uganda, suggesting that they are knowingly expecting hardships in their future rural practice.

This creates a new policy implication, that the government and nursing schools may want to court a particular "profile" of student associated with a lower tendency to emigrate and a higher sense of loyalty to the country when choosing whose education to subsidize or admit to nursing school. In our study, these were the students who wished to work in public practice or a rural area, the latter of which had no intention to emigrate. Students inclined towards public practice additionally believed that nursing students should stay in country if the country paid for the nurse's education. Government funding for nursing education could thus be prioritized towards these types of students, as opposed to being based solely on academic test scores. In this manner, governmental resources would be retained within country. Preferential admission based on a particular profile has been a strategy utilized for many years by numerous U.S. medical schools in an attempt to increase the number of physicians working in rural areas [21]. In South Africa, a study also found that rural-origin medical students were more likely to choose rural careers than urban-origin students. It recommended the selection criteria to be reviewed with regard to rural origin and career aspirations [22]. Admitting students with a commitment to rural areas in Uganda would meet the need for more nurses in rural areas as well as stem the number of nurses emigrating from the country as these are the students least likely to emigrate, based on our study.

## Conclusion

This paper is among the first to study nursing student perceptions towards emigration. It is also among the first to utilize a questionnaire in an attempt to quantify the importance of a push/pull factor. Among push/pull factors, students prioritized remuneration over all other factors, including job outlook, country stability or safety.

Students are attracted to public-sector work, although they perceive a shortage of public sector jobs. Government focus on providing more jobs and compensation in the public sector could be associated with a reduction in intent to migrate.

Students inclined towards rural practice or the public sector are less likely to desire emigration and express a higher sense of loyalty to their country. Their recruitment could lead to a more stable workforce in Uganda by increasing the number of nurses who choose to stay in Uganda as well as the numbers that work in rural areas. Therefore, nursing schools could use interviews, recommendations and personal goal statements in the admission process to favour those candidates likely to express a commitment to rural practice or continued service to Uganda. Government subsidy of nursing education could also be directed towards these students.

The U.S. was preferred over the U.K. as a destination, largely because the U.S. was perceived to have better remuneration and reduced competition for entrance into the country. This may mark a change in the traditional trajectory of most migrants to the U.K., and challenges the U.S. to evaluate its nursing workforce recruitment policies, with a goal to become self-sustaining in its nurse supply production [5].

## Competing interests

The author(s) declare that they have no competing interests.

## Authors' contributions

Lisa Nguyen and Steven Roper conceived the project and conducted the data collection. Nguyen conducted data entry and analysis, and wrote the paper. Esther Nderitu and Anneke Zuyderduin assisted with data collection at Aga Khan University. Sam Luboga provided faculty guidance at Makerere University. Hagopian provided continuing oversight and guidance to the project, from data collection to data entry to analysis and writing.
